# Molecular Signatures and Their Clinical Utility in Pancreatic Neuroendocrine Tumors

**DOI:** 10.3389/fendo.2020.575620

**Published:** 2021-01-18

**Authors:** Praveen Dilip Chatani, Sunita Kishore Agarwal, Samira Mercedes Sadowski

**Affiliations:** ^1^ Endocrine Surgery Section, Center for Cancer Research, National Cancer Institute, National Institutes of Health, Bethesda, MD, United States; ^2^ Metabolic Diseases Branch, National Institute of Diabetes and Digestive and Kidney Diseases, National Institutes of Health, Bethesda, MD, United States

**Keywords:** molecular signatures, signaling pathway, tumorigenesis, clinical trial, neuroendocrine carcinoma, pancreatic neuroendocrine tumor

## Abstract

Pancreatic neuroendocrine tumors (PNETs) are classified based on their histologic differentiation and proliferative indices, which have been used extensively to determine prognosis. Advances in next-generation sequencing and other high-throughput techniques have allowed researchers to objectively explore tumor specimens and learn about the genetic alterations associated with malignant transformation in PNETs. As a result, targeted, pathway-specific therapies have been emerging for the treatment of unresectable and metastatic disease. As we continue to trial various pharmaceutical products, evidence from studies using multi-omics approaches indicates that clinical aggressiveness stratifies along other genotypic and phenotypic demarcations, as well. In this review, we explore the clinically relevant and potentially targetable molecular signatures of PNETs, their associated trials, and the overall differences in reported prognoses and responses to existing therapies.

## Introduction

Neuroendocrine tumors (NETs) are a subset of neoplasms arising from neuroendocrine cells throughout the body that are characterized by their ability to produce peptides causing distinctive hormonal syndromes. Pancreatic neuroendocrine tumors (PNETs) comprise 1% to 3% of all newly diagnosed pancreatic cancers with an annual incidence of approximately 3.6 per 100,000 persons (1, 2). Though they represent only 7% of all gastrointestinal NETs, they are significantly more likely to present with distant metastases, at a higher grade, or with unresectable disease (~65%) compared to their stage-matched lung and small bowel counterparts (3, 4).

The prognosis of NETs is highly dependent on their histologic presentation. Median survival ranges from as low as 24 months in advanced, unresectable disease to seven years in early stage, resectable disease (5). In 2017, the World Health Organization established a new classification system utilizing grade (G1-G3, based on mitotic figures and Ki-67 index) and histologic differentiation (well-differentiated “neuroendocrine tumors” vs. poorly differentiated “neuroendocrine carcinomas”) (**Table 1**) (6). This system aims to stratify tumors by aggressiveness, scale treatment accordingly, and guide discussions regarding prognosis. The update was prompted by significant disparities in patient survival when classified by grade alone. While G3 tumors comprise only ~10% of PNETs, they occupy a spectrum of aggressiveness depending largely on their histologic differentiation (7–10).

**Table 1:** World Health Organization classification and grading of pancreatic neuroendocrine neoplasms (2017) (6).

Differentiation and Grade	Proliferation Index Ki-67 (%)	Mitotic Index (Mitoses per 10 HPFs)
**Well-Differentiated** • **G1 PNET** • **G2 PNET** • **G3 PNET**	<3 3–20 >20	<2 2–20 >20
**Poorly Differentiated** ** • G3 PNEC**	>20	>20

PNET, Pancreatic Neuroendocrine Tumor.

PNEC, Pancreatic Neuroendocrine Carcinoma.

HPFs, High power fields.

Although functionality is not considered in the classification, the vast majority of PNETs (85%) are clinically non-functional and are often incidentally discovered during abdominal imaging (~40%) (11, 12). The clinical presentation of non-functional PNETs is due to mass-related burden of the primary tumor or metastatic deposits (up to 50% have metastatic disease upon presentation) (13). For this reason, they are often diagnosed at more advanced stages and are associated with poorer prognosis. Their median overall survival (OS) is 26 months versus 54 months in their functional counterparts (14–16).

PNETs are most commonly sporadic, but they may also occur as part of five hereditary syndromes: multiple endocrine neoplasia type 1 (MEN1), MEN4, von Hippel-Lindau (VHL), neurofibromatosis type 1 (NF1), and tuberous sclerosis complex (TSC). Together, these syndromes account for less than 10% of PNETs and, while they are more likely to present with multi-focal disease, their rate of metastasis is slightly lower at 23% to 33% of cases (16, 17). Among the hereditary syndromes, functioning PNETs such as insulinomas, gastrinomas, glucagonomas, VIPomas, and somatostatinomas are observed most frequently in MEN1 (18, 19).

Amidst this wide array of severities, surgery remains the cornerstone of treatment for this disease and should be pursued for all local or metastatic neoplasms amenable to complete resection (20–22). Presently available systemic options are limited to long-acting release octreotide, the mTOR inhibitor everolimus, and the VEGF inhibitor sunitinib (23–25). These drugs offer a modest improvement in progression-free survival but have not been shown to impact OS. Despite the relative success of surgery, up to 50% of patients who undergo complete resection will develop metachronous liver metastases and, although recurrence-free survival is positively associated with well-differentiated tumors, it is evident that the current characterization model does not successfully separate biologically favorable tumors from those with a predilection for recurrence/metastasis (12).

Advances in next-generation sequencing have allowed researchers to objectively explore tumor specimens and learn about the genetic alterations that drive malignant transformation in PNETs. Analyses of this molecular landscape have identified significant heterogeneity in the mutational profiles of non-functional PNETs. However, four core pathways have been implicated: DNA damage/repair, chromatin remodeling, alternative lengthening of telomeres, and PI3K/AKT/mTOR signaling (26). Among the functional tumors, genetic and expression profiling of insulinomas did not show mutations in *MEN1*, *DAXX/ATRX*, and mTOR pathway genes that are frequently mutated in non-functional PNETs (27). Approximately 3% to 30% of insulinomas show a recurrent mutation in *YY1* (p.T372R), a gene encoding a transcription factor (28–30). Also associated with insulinomas are rare mutations or gene expression changes of a few epigenetic modifier genes (*H3F3A*, *KDM6A*, *ATR*, and *EZH2*) (27). The molecular patterns of presentation that have been identified in PNETs can help us predict clinical outcomes.

This review will explore the clinically relevant and potentially targetable mutational and immunohistochemical features, henceforth referred to as “molecular signatures”, of non-functional PNETs, their associated trials, and overall differences in reported prognoses.

## Molecular Mechanisms and Prognosis in PNETs

**Figure 1:**
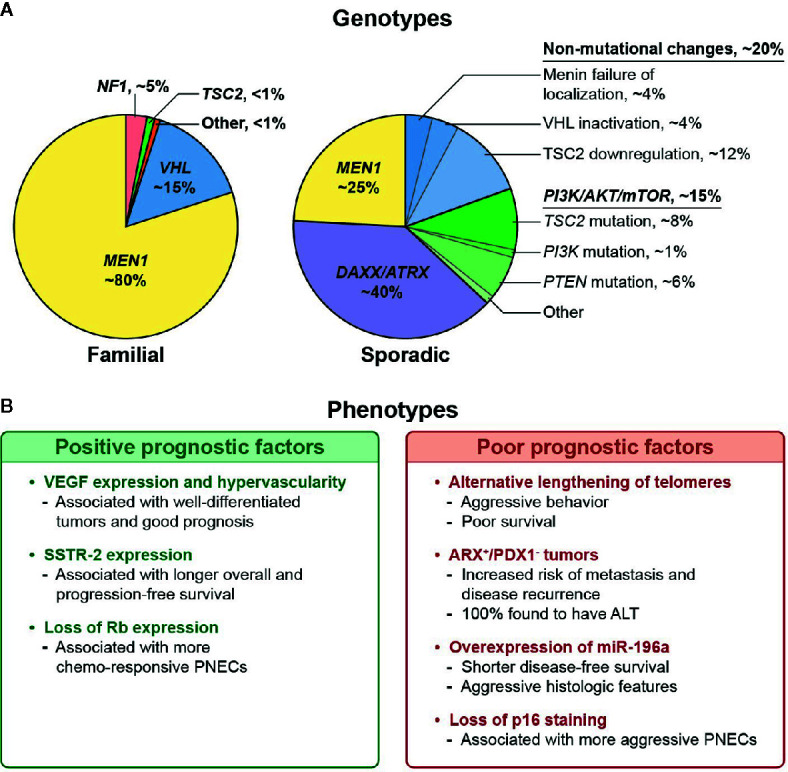
**(A)** Reported distribution of genotypic alterations in familial and sporadic pancreatic neuroendocrine tumors (PNETs). **(B)** Prognostic associations of various non-functional PNET phenotypes. (11, 14, 31–34).

Inherited tumor syndromes, including MEN1, VHL, NF1, and TSC, represent a minority of PNETs, but uncovering their molecular mechanisms has furthered our understanding of their sporadic counterparts. Somatic mutations or alterations of the relevant syndromic genes are frequently seen in sporadic PNETs. Up to 44% of non-familial PNETs harbor a somatic *MEN1* alteration, while 25% have non-mutational VHL inactivation, and downregulation or mutation of the *TSC2* gene is seen in 35% and 9%, respectively (26). This supports the notion of shared pathways of tumorigenesis in familial and non-familial tumors centered around the *MEN1* gene (**Figure 1**). Further complicating matters, germline mutations in genes linked to familial syndromes (i.e., MEN1, VHL) have also been detected in patients with apparently sporadic PNETs (35). In this section, we explore the complex molecular landscape of PNETs as it relates to prognosis.

### *MEN1*

Menin, the gene product of *MEN1*, is a predominantly nuclear protein that functions as a tumor suppressor by i) interacting with various transcription factors and histone modification enzymes to regulate gene transcription, ii) regulating cell cycle progression (*via* upregulation of CDKN2C/CDKN1B expression), and iii) participating in specific cell signaling processes (for example, menin alters AKT1 sub-cellular localization to regulate the PI3K/AKT/mTOR signaling pathway) (26). Patients with MEN1 (a heterozygous germline mutation in the *MEN1* gene inherited in an autosomal-dominant fashion) have a 40%–80% chance of developing a PNET during their lifetime, making it the second-most-frequently expressed clinical manifestation of the syndrome next to parathyroid neoplasms (36). These are most commonly non-functional PNETs with a tendency towards multiplicity. Tumor size at the time of presentation is also an important prognostic factor, as lesions > 2 cm harbor a higher risk of malignancy (17, 37). Tumors show loss of heterozygosity at the *MEN1* locus on chromosome 11q13 and abnormally low nuclear staining of menin (38, 39). While this is the established mutational mechanism in familial cases, a 2010 combined genetic and immunohistochemical study of *MEN1* mutations and menin expression demonstrated that up to 80% of sporadic cases had strong cytosolic staining of the protein, suggesting a failure of nuclear localization, while just 25% of the patients harbored a mutation in the gene itself (31). This suggests that other pathways or genes are involved in the altered expression of menin or its downstream effects. Directly or indirectly, *MEN1* and its associated pathways play an important role in the neoplastic process of PNETs and represent potential therapeutic targets. Among sporadic PNETs, 44% of non-functional tumors show *MEN1* mutations, and the prevalence of a *MEN1* mutation in functional tumors is as follows: glucagonoma (60%), VIPoma (57%), gastrinoma (38%), and insulinoma (2%–19%) (40).

### *VHL*

Contrary to MEN1, VHL-associated PNETs are reported in just 10%–17% of syndromic patients, most of which present at a young age and with non-functional, multi-focal disease throughout the pancreas (32, 41–44). Although *VHL* mutations are rarely seen in sporadic PNETs, non-mutational *VHL* inactivation is seen in up to a quarter of sporadic PNETs. Compared with sporadic tumors, though, resected VHL-associated PNETs have better long-term outcomes (45). VHL-driven PNETs likely represent a distinct subset of these tumors. Unlike their *MEN1* or *DAXX/ATRX* mutation-positive counterparts, genetic alterations in *VHL*-mutation driven PNETs are predominantly related to angiogenesis and hypoxia-inducible factor (HIF) signaling (46). The VHL protein itself serves as a regulator of HIF-1α. Thus, inactivation of *VHL* induces angiogenesis and abnormal cell metabolism. A quantitative RT-PCR study of 52 genes in 18 patients with VHL-associated PNETs demonstrated a unique pattern of gene upregulation related to HIF signaling, angiogenesis, and specific growth factor/cell cycle component expression when compared to sporadic tumors (46).

### *HIF-1α* and *VEGF*

Whether sporadic or familial, PNETs are highly vascularized owing to increased levels of proangiogenic factors such as HIF-1α and vascular endothelial growth factor (VEGF). The HIF-1 pathways regulate over 100 genes involved in angiogenesis including *VEGF*, *PDGF*, and angiopoietin (*ANG-1* and *ANG-2*) (47). VEGF itself is expressed in ~80% of PNETs. Well-differentiated PNETs have been shown to express higher levels of these factors and have a higher density of microvasculature than poorly differentiated PNETs (48, 49). Surprisingly, this higher microvessel density has been associated with a better prognosis (50). Immunostaining of tumor sections from 45 patients with PNETs showed that well-differentiated tumors had high cytoplasmic expression of VEGF and HIF-1α, whereas poorly differentiated carcinomas were associated with nuclear HIF-1α expression. Also, nuclear HIF-1α expression was associated with the presence of necrosis, larger tumor size, low microvascular density, and shorter survival (49). Insights into the mechanisms underlying angiogenesis have led to the development of VEGF inhibitors such as bevacizumab and sunitinib that are being tested as potential PNET therapies (51, 52). Recently, a Phase Ib/II trial of surufatinib, a selective VEGF receptor (VEGFR) small molecule tyrosine kinase inhibitor, demonstrated encouraging antitumor activity with minimal toxicities in patients with advanced NETs (53).

### *DAXX/ATRX*

Up to 43% of sporadic PNETs have a mutation in genes encoding for a chromatin remodeling/stabilization complex composed of death domain-associated (DAXX) protein and α-thalassemia/intellectual disability syndrome X-linked (ATRX) protein (33). This heterodimer is implicated in remodeling at the telomeric and pericentromeric regions, and incorporating the histone variant H3.3, a mechanism supported by nuclear staining of the proteins in *DAXX/ATRX* wild-type PNETs that is absent in PNETs with mutant proteins (14, 54–56). Mutations in *DAXX/ATRX* are mutually exclusive, can promote tumorigenesis, and correlate with alternative lengthening of telomeres (ALT), a telomerase-independent mechanism of telomere lengthening (11). This phenotypic abnormality was found in 61% of patients in a molecular analysis of PNETs by Heaphy et al. The study demonstrated a significant correlation between either *ATRX/DAXX* mutations or the loss of their respective nuclear proteins and the presence of ALT across multiple tumor histologies (33). Though this genotype–phenotype relationship is strong, it is present in only ~3% of all human neoplasms. Regardless, it represents a clinically significant marker, as research predicts that ALT indicates resistance to anti-telomerase therapies and may harbor prognostic value (57).

Despite being exclusively linked to well-differentiated PNETs, *ATRX/DAXX*-mutated PNETs have been clinically associated with aggressive behavior and poor survival (58). A large retrospective study attributed this poor survival to increased chromosomal instability seen in patients with *DAXX/ATRX*-mutated PNETs (59). Clinicopathologic and genetic analysis of 76 patients with well-differentiated PNETs in South Korea showed DAXX/ATRX loss to be independently associated with poor prognosis alongside metastatic disease on presentation (60). Telomere-specific fluorescence *in situ* hybridization (FISH) analysis of 109 well-differentiated PNETs only identified *DAXX/ARTX* mutations and ALT phenotype in patients with tumors greater than 3 cm and lymph node metastases, suggesting that these changes may be specific to the later stages of disease (61). Similarly, in multiple independently examined cohorts of surgically resected PNETs, ALT-positive tumors displayed a significantly higher grade, size, and pT staging. ALT phenotype and loss of DAXX/ARTX staining correlated strongly (*P* < 0.05) with lymphovascular invasion, perineural invasion, lymph node involvement, distant metastasis, and shorter recurrence-free survival (62, 63).

### *PI3K/AKT/mTOR*

Expression profiles of sporadic PNETs have shown a correlation between prognosis and the PI3K/AKT/mTOR pathway that is involved in the regulation of cellular proliferation, growth, survival, and other vital functions (64). Exome sequencing of PNETs has shown that ~15% of well-differentiated tumors harbor somatic mutations in the PI3K/AKT/mTOR pathway genes, including 7% in *PTEN*, 9% in *TSC2*, and 1% in *PIK3CA* (14). Whole-genome sequencing of 102 sporadic PNETs found *PTEN* and *TSC1/2* mutations to be mutually exclusive (35). As it is central to multiple tumorigenic pathways, downregulation of mTOR pathway inhibitors such as PTEN and TSC2 was a highly significant finding (~85%) in a gene expression profiling of PNETs, even in the absence of a pathway-specific mutation. Under-expression of these essential regulatory factors was associated with more-advanced stage, increased risk of metastasis, and shortened disease-free survival and overall survival (11, 64).

### Chromosomal Aberrations

Mutations alone provide the rationale for tumorigenesis in only about 40% of sporadic PNETs, with the remaining cases ascribed to chromosomal/epigenetic alterations (26). Various studies have identified non-mutational biomarkers associated with tumor progression/metastasis that could potentially assist with management. Copy-number alterations at certain chromosomal loci, such as 6p22.2-p22.1, 8q24.3, 9q34.11, and 17p13.1 have been associated with poorer prognosis (65). This is an important area of ongoing research, though the prognostic implications of other copy-number alterations and chromosomal translocations are poorly defined at this time.

### Micro-RNAs and DNA Methylation

miRNAs are short, non-coding RNA molecules regulating gene expression at the post-transcriptional level; they have been shown to regulate up to 60% of all coding genes and have been implicated in a wide range of biologic processes, including carcinogenesis (66). A micro-array study of 44 pancreatic primary tumors, including functional and non-functional PNETs, discovered patterns of miRNA expression that could distinguish any tumor type from normal pancreas (67). For example, expression of *miR-103* and *miR-107* in the absence of *miR-155* distinguished tumors from normal pancreas. miRNA expression patterns were able to distinguish PNETs from acinar carcinomas and further segregate PNETs by functionality, Ki-67 index, and presence of liver metastases. More recently, a comparison of miRNA and mRNA transcriptomes in PNET samples showed an association between miRNA profiles and commonly described genetic alterations, such as *DAXX/ATRX* and *MEN1* (68). Over-expression of *miR-196a* in post-resection PNETs has been associated with poor prognosis, shorter disease-free survival, and more aggressive histologic profile (stage, mitotic rate, Ki-67) (69). There is mounting evidence that miRNA expression profiles may serve as a clinically useful biomarker for diagnostic, prognostic, and therapeutic purposes (70).

DNA methylation profiles of PNETs may also serve as relevant biomarkers for tumor stratification. Hyper-methylation or hypo-methylation at CpG islands (GC-rich DNA regions), often located in the promoter regions of genes, is associated with gene silencing or gene activation, respectively. Higher global DNA hypermethylation was detected in non-functional PNETs from MEN1 patients in comparison with sporadic and VHL-associated PNETs (71). Also, promoter hypermethylation was observed as a frequent event in MEN1-associated advanced PNETs (72). Validation of these findings in independent cohorts of familial and sporadic PNETs will help to determine the importance of DNA methylation as a biomarker.

### Circulating Tumor DNA/RNA

Minimally invasive liquid biopsy tests for advanced cancers using blood/plasma have been shown to detect tumor-specific mutations in circulating cell-free tumor DNA (ctDNA), and recent data from studies of the NETest have shown successful detection of tumor-specific transcripts in circulating RNA (73, 74). Genç et al. demonstrated that this multigene blood test could effectively detect PNET recurrence after surgical resection (test performed after recurrence) in a cohort of non-functional (83%) and functional (17%) PNETs (75). A recent meta-analysis shows an accuracy of 90.2%–93.6% as a marker of natural history of NET (76). However, large validation studies with long-term follow-up are needed. The NETest could help in the detection of sporadic PNETs or their response to therapy; however, the utility of the NETest to detect PNETs or their metastases in patients presenting with familial syndromes could be confounded by the simultaneous presence of other tumors.

### Cell of Origin and Transcription Factors

The clinical behavior of non-functional PNETs is also correlated with the transcriptomic profiles of specific islet cell types, namely α- and β-cells that undergo differentiation *via* transcription factors ARX and PDX1, respectively. A study of 142 non-functional PNETs (MEN1-associated and sporadic) showed that 84% of tumors expressed either ARX or PDX1, predominantly, and could be classed as “A-type” (resembling α-cells) or “B-type” (resembling β-cells) (34). Of the 103 tumors with subsequent distant relapses, almost all were ARX^+^PDX1^-^ and had ALT. The authors postulated that this molecular stratification could provide insight into the correlation of cell lineage with disease course and inform postoperative clinical decisions.

### Unique Molecular Mechanisms in Pancreatic Neuroendocrine Carcinomas

To this point, this review has focused on molecular alterations more common in well-differentiated pancreatic neuroendocrine tumors. It should be noted that poorly differentiated pancreatic neuroendocrine carcinomas (PNECs), which carry significantly worse prognoses, represent a genetically distinct biology by comparison. PNECs often harbor alterations in *Rb* and *TP53* (~74% and 95%, respectively). An immunostaining study showed that inactivation of these two pathways is a central feature of PNEC development (58). Aberrant p16 staining was found to be mutually exclusive to Rb anomalies, such that all PNECs had some disruption of the p16/Rb pathway. BCL2 overexpression, while seen in both PNEC (74%) and PNET (18%) samples, was much more common in the former. In contrast, SMAD4/DPC4, DAXX, and ATRX staining was found to be intact in all samples (58). Though overall prognosis across all PNECs is dismal, subtle differences in molecular signatures have demonstrated overall differences in prognosis. A study assessing response to platinum-based chemotherapy showed that tumors with loss of Rb expression had a significantly better response to treatment (80% vs 38.4%) than those that retained Rb; a similar improvement in overall response was seen in patients with KRAS mutations compared to those with KRAS wild-type tumors (77% vs. 23%) (77). Such distinct molecular signatures aid in characterizing G3 tumors as PNETs or PNECs, thereby delivering potentially accurate prognostic information and, more importantly, tailored therapeutic regimens to patients. As discussed below, recommended treatments by the European Neuroendocrine Tumor Society and the National Comprehensive Cancer Network differ for G3 PNECs (78, 79).

## Therapeutic Advances and Implications of Molecular Signatures in PNET/PNEC

In patients with resectable disease, R0/R1 resection remains the treatment of choice. Unfortunately, the majority of patients with PNETs either present with or develop metastatic disease within two years of surgery, prompting the need for effective systemic therapies (80). So far, there are a number of standard-of-care therapies available, including somatostatin analogues, cytotoxic chemotherapies, inhibitors of the angiogenesis and mTOR pathways, and combinations of multi-drug regimens (**Table 2**). There are, in addition to established treatment regimens, a number of drugs in clinical trials. However, there is a paucity of data focused on directing these therapies to their most appropriate candidates.

**Table 2:** Available therapeutic agents and their respective targets.

Molecular Pathway	Target	Available Therapeutic	FDA Approval	Outcomes (vs. placebo/control)	Source
*Angiogenesis*	VEGF	Bevacizumab Pazopanib			
	+PDGF	Sunitinib	*	ORR: 9% Median PFS: 12.6mo (vs. 5.8)	[81, 82]
		Sorafenib			
	+cMet	Surufatinib	*	ORR: 19% Median PFS: 21.2mo	[53]
*Somatostatin*	SSTR-2	Octreotide	*	Median OS: 77.4mo (vs. 73.7mo) Median PFS: 14.3mo (vs. 6mo)	[24]
		Lanreotide	*	Median PFS: NR (vs. 18mo) PFS at 24mo: 65% (vs 33%)	[25]
		PRRT (^177^Lu-Dotatate)	*	ORR: 18% (vs. 3%) PFS at 20mo: 65% (vs. 11%)	[83]
	+SSTR-1, 3 and 5	Pasireotide			
*PI3K/AKT/mTOR*	mTOR	Everolimus Temsirolimus	*	Median OS: 44mo (vs. 37.7mo)	[84]
*Immunomodulation*	PD-1	Pembrolizumab	**	Not Done	

ORR, Overall response rate.

PFS, Progression-free survival.

OS, Overall survival.

*Approved for NETs

**Approved for other solid tumors.

### Somatostatin Receptor Expression and Blockade

Neuroendocrine tumors typically express somatostatin receptors on their cell membranes, which have demonstrated diagnostic, prognostic, and therapeutic utility. Recently, 68-Gallium-DOTATATE positron emission tomography demonstrated diagnostic superiority to conventional functional imaging modalities while more accurately identifying clinically aggressive and treatment-refractory tumors (85, 86). Of the five different somatostatin receptor (SSTR) subtypes, SSTR-2 is expressed on approximately 80% of PNETs, making it an attractive target for therapeutic intervention (87). While the ability to mitigate the effects of hormonal over-secretion is, in and of itself, useful, objective tumor shrinkage with somatostatin analogues is rare, and these drugs have predominantly been used to slow the pace of disease. The phase III CLARINET trial, which compared lanreotide to placebo in 204 patients with non-functioning, SSTR-expressing gastrointestinal NETs (45% of which were pancreatic), showed a significantly prolonged progression-free survival (PFS) with SSTR blockade (25). Based on this data, lanreotide has been approved by the U.S. Food and Drug Administration (FDA) for the treatment of patients with unresectable, well- or moderately differentiated, locally advanced or metastatic gastroenteropancreatic NETs. A study examining the use of lanreotide in NETs postulated that PNETs may fall among the histologies least likely to respond, though there are no studies that stratify the responses by molecular characteristics (88). More recently, the β-radiation-emitting compound ^177^Lu-Dotatate, a radiolabeled somatostatin analog, was approved for systemic treatment of inoperable or metastatic disease after demonstrating improved PFS and a significantly higher response rate compared to high-dose octreotide LAR (83).

### Angiogenic Factor Expression and Inhibition

A deeper understanding of the molecular features of PNETs, such as their abundant intra-tumoral vasculature and expression of angiogenic factors, has led to the application of targeted therapies like anti-VEGF and anti-PDGF drugs (89). Sunitinib, a potent antagonist of VEGF and PDGF signaling, demonstrated an overall response rate of 9%, with median PFS of 12.6 months versus 5.8 months in placebo-controlled patients, leading to its FDA approval for unresectable, locally advanced or metastatic PNETs (81, 82). Other VEGF inhibitors such as bevacizumab have shown similar overall response rates to monotherapy but have demonstrated greater utility as a component of a multi-drug regimen (52). Inhibition of VEGF signaling was, in some pre-clinical models, associated with increased invasiveness and metastasis attributed to the over-expression of c-Met. Subsequent trials examining blockade of this increased c-Met signaling alongside VEGF inhibition identified it as a potential escape mechanism for patients on anti-angiogenic therapies (90). Overexpression of c-MET alone has also been detected in PNETs, making it a therapeutic target in need of further study (91). Drugs such as surufatinib—a small-molecule tyrosine kinase inhibitor of VEGF, fibroblast growth factor, and CSF receptors—aim to target multiple angiogenic pathways simultaneously; encouraging phase Ib/II data for surufatinib has prompted ongoing phase III studies (53). Though a number of studies have examined baseline levels and treatment-related changes in soluble plasma and tumor tissue markers such as VEGF and its associated receptors, none have identified a clinically reliable treatment (92).

### mTOR Pathway Dysregulation and Inhibition

A landmark phase III trial called RADIANT-3 showed a 6.4-month improvement in median PFS with an mTOR inhibitor called everolimus but was unable to demonstrate an OS advantage (23, 84). A prognostic effect has been observed with lower baseline levels of and treatment-related reduction in chromogranin A, neuron-specific enolase, placental growth factor, and soluble vascular endothelial growth factor receptor 1 (84, 93, 94). Several studies have demonstrated mechanisms of resistance responsible for the underwhelming effect of the drug and, although combining mTOR inhibitors with other PI3K/AKT inhibitors and/or anti-angiogenic drugs has proven superior to monotherapy, an organized effort to identify molecular sub-groups with more promising responses has yet to be undertaken (52, 95–98). Previous studies have shown that the presence of certain *PI3K* and *KRAS* mutations may influence breast cancer cells’ response to everolimus. Meanwhile, deletion of the KRAS mutation restores sensitivity to the therapy (99). Studies examining molecular predictors of response to mTOR inhibition also demonstrated increased efficacy in tumors with a higher percentage of phosphorylated (i.e., activated) mTOR, as assessed by immunohistochemistry (100, 101). So far, confirmation of a PI3K/AKT/mTOR pathway genetic mutation has not precluded inclusion in any trial of mTOR pathway inhibitors in PNETs.

### Checkpoint Receptor Expression and Blockade

Finally, no discussion of contemporary systemic cancer therapies is complete without mentioning immune checkpoint inhibitors. Although PD-1 and PD-L1 expression in PNETs is rare, PD-L2 and abundant T-cell infiltrates have been noted when characterizing the immune microenvironment, suggesting a potential role for checkpoint blockade (102). No association has been seen, however, between T-cell infiltration and aforementioned molecular/mutational signatures in PNET, and further studies are warranted to determine the existence of such links. Initial data from the KEYNOTE-028 study (NCT02054806) showed a 3.7% response rate, with only one responder being a patient with PNET (103).

### Cytotoxic Chemotherapies and Combination Approaches

Various studies have demonstrated that PNETs are responsive to cytotoxic chemotherapy. Streptozocin, an alkylating agent, was approved by the FDA in the early 1980s for the treatment of neuroendocrine carcinomas, when it achieved a 69% radiologic response rate and median survival of 2.2 years when administered in conjunction with doxorubicin (104). Concerns surrounding inconsistencies in the reproducibility of these data, toxicity, and the drug’s cumbersome infusion schedule have made streptozocin a less-popular agent in the treatment of PNETs (105). Subsequent studies led to increased use of similar alkylating agents like dacarbazine and, more recently, temozolomide, which has produced response rates up to 70% when delivered in conjunction with capecitabine (106). More recent prospective trials of temozolomide alongside targeted agents such as bevacizumab and everolimus have produced response rates of 33% and 40%, respectively (107, 108). Objective response rates of temozolomide-based therapy in PNEC are similarly unsatisfying at 33% (109). Patients who lack a certain DNA repair enzyme conferring resistance to temozolomide (O^6^-methylguanine DNA methyltransferase or ‘MGMT’), or who have reduced expression of MGMT due to promoter methylation, see greater efficacy of the drug than those with increased expression (110, 111); aside from this, there are no studies examining molecular factors predictive of response to cytotoxic chemotherapy (26, 112).

The majority of clinical studies with respect to PNET therapies examine small cohorts of patients from single institutions with a lack of consideration given to molecular signature or lack of syndromic patients. Trials of everolimus do not segregate patients based on genetic integrity of their mTOR pathway components, and those examining anti-angiogenic medications are not stratified based on upregulation of anti-angiogenic factors themselves. Moving forward, it is important that these molecular signatures be taken into greater consideration when evaluating the impact of therapeutic approaches. As is often suggested, this will require further multi-institutional collaboration bridged by common nomenclature and classification systems.

## Precision Oncology and the Role of Molecular Signature

A few recent studies have used a precision oncology approach to select drugs and druggable targets in gastroenteropancreatic (GEP) NETs. RNA-seq-derived gene expression patterns in matched primary PNETs and their metastases from 43 patients were analyzed to identify metastasis-associated pathways (113). Metastasis-specific alterations were identified in MAPK, cyclin-dependent kinase (CDK), topoisomerase (TOP2A), NF-kB, and PI3K/mTOR signaling pathways. This study provides valuable insights into potentially druggable targets for the pre-clinical assessment of pathway-specific drugs to treat advanced PNETs. Another study used RNA-seq analysis of 212 GEP-NET samples (including 83 PNETs) followed by virtual inference of protein activity to identify master regulators that represented highly enriched tumor-essential genes (114). A similar analysis in GEP-NET cell lines was coupled with systematic drug perturbation assays using 107 small molecule compounds that could invert specific master regulator protein activity signatures (tumor checkpoint collapse) (114). Entinostat, an HDAC class I inhibitor, demonstrated cytotoxicity and reversal of master regulatory protein activity signatures in DEP-NET cell line models and predicted efficacy in 42% of tumor specimens (with similar master regulator protein activity signatures). This study shows how to prioritize drugs for preclinical assessment that target specific tumor checkpoints. The choice of cell line models is critical for the success of these precision oncology approaches. Whole-exome sequencing, copy-number analysis, and immunophenotyping of NET cell lines has allowed for a deeper understanding of NET cell line model systems and facilitated *in vitro* testing of various therapeutics on different NET phenotypes (113, 115).

## Conclusions

Despite significant progress in the characterization of PNETs at the histologic and molecular level, clinically correlative findings have largely been limited to prognostication. This is largely due to the fact that clinical trials are not subject to mutation-specific inclusion criteria. For years, these tumors have been classified by their histologic differentiation and Ki-67 proliferative index. However, it is clear that clinical aggressiveness also stratifies along other genotypic/phenotypic lines. Therefore, future studies must consider important tumor-specific genetic, epigenetic, and transcriptomic alterations before and after therapy, such as MEN1/*DAXX/ATRX* mutations, ARX expression, and their corresponding phenotypic manifestations (i.e., ALT), as defining characteristics that may impact tumor recurrence and the survival of patients with PNETs. As we move into an era of personalized medicine, where next-generation sequencing is more readily and widely available, prior ‘one-size fits all’ models of classification must be replaced by more-informed systems.

## Author Contributions

PC and SS: Conception of the work and drafted the article. SS and SA: Critical revision of the article. All authors contributed to the article and approved the submitted version.

## Funding

Funding support for this review was provided by the Intramural Research Program of the National Institutes of Health: NCI (SS and PC) and NIDDK (SA). ZIA DK075035-12 for SA.

## Conflict of Interest

The authors declare that the research was conducted in the absence of any commercial or financial relationships that could be construed as a potential conflict of interest.
